# Assessing the efficacy of CRISPR/Cas9 genome editing in the wheat pathogen *Parastagonspora nodorum*

**DOI:** 10.1186/s40694-020-00094-0

**Published:** 2020-03-31

**Authors:** Haseena Khan, Megan C. McDonald, Simon J. Williams, Peter S. Solomon

**Affiliations:** Division of Plant Sciences, Research School of Biology, The Australian National University, Canberra, 2601 Australia

**Keywords:** CRISPR/Cas9, Gene editing, Ribonuclear protein complex, *Parastagonospora nodorum*

## Abstract

**Background:**

The genome-editing tool CRISPR/Cas9 has revolutionized gene manipulation by providing an efficient method to generate targeted mutations. This technique deploys the Cas9 endonuclease and a guide RNA (sgRNA) which interact to form a Cas9-sgRNA complex that initiates gene editing through the introduction of double stranded DNA breaks. We tested the efficacy of the CRISPR/Cas9 approach as a means of facilitating a variety of reverse genetic approaches in the wheat pathogenic fungus *Parastagonospora nodorum*.

**Results:**

*Parastagonospora nodorum* protoplasts were transformed with the Cas9 protein and sgRNA in the form of a preassembled ribonuclear protein (RNP) complex targeting the *Tox3* effector gene. Subsequent screening of the *P. nodorum* transformants revealed 100% editing of those mutants screened. We further tested the efficacy of RNP complex when co-transformed with a *Tox3*-Homology Directed Repair cassette harbouring 1 kb of homologous flanking DNA. Subsequent screening of resulting transformants demonstrated homologous recombination efficiencies exceeding 70%. A further transformation with a *Tox3*-Homology Directed Repair cassette harbouring a selectable marker with 50 bp micro-homology flanks was also achieved with 25% homologous recombination efficiency. The success of these homology directed repair approaches demonstrate that CRISPR/Cas9 is amenable to other in vivo DNA manipulation approaches such as the insertion of DNA and generating point mutations.

**Conclusion:**

These data highlight the significant potential that CRISPR/Cas9 has in expediting transgene-free gene knockouts in *Parastagonospora nodorum* and also in facilitating other gene manipulation approaches. Access to these tools will significantly decrease the time required to assess the requirement of gene for disease and to undertake functional studies to determine its role.

## Background

*Parastagonospora nodorum* is a devastating pathogen of wheat that impacts on yields globally. *P. nodorum* is a causative agent of Septoria nodorum blotch (SNB) and is responsible for in excess of $100 million AUD in wheat yield losses each year in Australia alone [1]. To better understand the mechanistic basis of disease, fundamental research has been undertaken with a focus on discerning the molecular interactions between the plant and pathogen. These studies have identified and characterized several of the factors that *P. nodorum* deploys to cause disease and have led to a recognition and understanding of the specific gene-for-gene interactions that underpin SNB [2].

A key aspect to this success was the exploitation of gene deletion/disruption techniques that identified the requirement and function of a particular gene involved in disease. One of the limiting factors in efficiently disrupting genes in *P. nodorum* has traditionally been low frequencies of homologous recombination at a targeted locus [3]. A breakthrough that improved rates of homologous recombination in fungi evolved through the disruption of genes involved in the non-homologous end joining (NHEJ) pathway. Impairment of the KU70 gene (within the NHEJ pathway) substantially increased rates of homologous recombination in many, but not all, fungi [4–7]. Similarly, the NHEJ pathway has been targeted in *P. nodorum* resulting in a drastic increase in the efficiency of homologous recombination for the pathogen [8].

However, this approach is not without its drawbacks and inactivation of the NHEJ pathways has been associated with increased vulnerability to DNA damaging conditions [9–12]. Further improvements are also desirable, particularly in the efficiency and also time required for generating disruption constructs. There is also interest in developing marker-free mutants which is challenging with existing approaches. Other reverse genetic applications are also desired whereby finely resolved changes of the genome require more precise approaches than homologous recombination with a large selectable marker (e.g. introducing a single nucleotide polymorphism into promoters/genes). One such tool that can potentially facilitate many of these improvements is the recently described Clustered Regularly Interspaced Short Palindromic Repeats-Cas9 system (CRISPR/Cas9) system for genome editing. CRISPR/Cas9 is derived from the bacterial and archaeal immune system [13]. This gene editing system comprises of two components, a single guide RNA (sgRNA) and an endonuclease enzyme, Cas9. The sgRNA recognizes a protospacer sequence which then binds to the targeted DNA region upon base pairing and interacts with Cas9 to become a stable Cas9-sgRNA complex. Once recognition has occurred, Cas9 cleaves the double stranded DNA, creating a double stranded break (DSB), leading to an activation of DNA repair. It has been confirmed from previous studies that CRISPR/Cas9 could induce highly efficient mutagenesis via both HDR and NHEJ pathways in a range of eukaryotic systems [13].

The utility of CRISPR/Cas9 has now been demonstrated in numerous filamentous ascomycetes fungi including *Trichoderma reesei* [14], *Aspergillus nidulans* [15], *Aspergillus fumigatus* [16], *Neurospora crassa* [17], *Fusarium oxysporum* [18] and *Magnaporthe oryzae* [19]. In this study, we assessed the efficacy of CRISPR/Cas9 gene editing in *P. nodorum*. To do this, we chose the ribonuclear protein (RNP) complex approach to deliver the sgRNA and Cas9 into the fungus. The RNP complex is formed through a short incubation of the sgRNA and purified Cas9 protein [20] which is then transformed into fungal protoplasts. The RNP complex approach offers many benefits compared to others (e.g. expression of Cas9 and sgRNA inside the targeted cells). For example, introduction of the purified Cas9 protein and sgRNA avoids integration of genetic material in untargeted regions of the genome thereby reducing off-site effects [21]. Once inside the targeted organism, the RNP complex is rapidly degraded [22]. The RNP approach also provides the means of determining the efficiency of a designed sgRNA for targeting Cas9 to the targeted gene location through in vitro cleavage assay. This assay therefore provides confidence that the RNP complex can efficiently cleave the targeted gene.

In this study we measured the efficacy of CRISPR/Cas9 in *P. nodorum* using two different approaches. Firstly, we sought to mutate a targeted sequence by exploiting the unguided repair by the NHEJ pathway of a targeted DSB induced by Cas9 (known as SDN1) [23]. In a second approach, we attempted a template-guided repair of a targeted DSB using a homology directed repair (HDR) cassettes comprising of a selectable marker with flanks of double stranded DNA homologous to the targeted sequence (SDN3). These experiments provide an important step in understanding the possibilities and limitations of gene editing in this important pathogen.

## Results and discussion

### The wheat pathogen *P. nodorum* is amenable to CRISPR/Cas9 genome editing

**Fig. 1:**
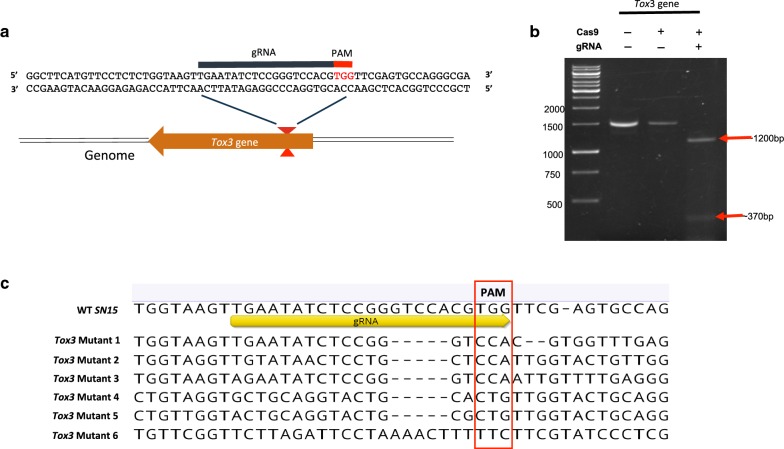
CRISPR/Cas9 genome editing of *Tox3* in *P. nodorum* via delivery of *Tox3*-RNP complex. **a** A sequence schematic highlighting the sequence of the gRNA and location of the targeted site in the *Tox3* gene. **b** A gel image demonstrating the in vitro cleavage of *Tox3* in the presence of the *Tox3*-RNP complex. Bands of 1200 bp and 370 bp are consistent with the sizes expected for the targeted site within *Tox3* after cleavage. **c** Sequence alignments of the targeted *Tox3* region in selected mutants. The sequences demonstrate a variety of SNPs and small indels consistent with the activity of Cas9 at the targeted site. All strains screened were mutated implying a 100% efficiency of editing at the *Tox3* locus

A suitable sgRNA sequence for *Tox3* was synthesized as described below and used to form an RNP complex with Cas9 through a brief co-incubation at room temperature [24] (Fig. 1a). An in vitro cleavage assay of the *Tox3* gene with the RNP complex generated the expected size bands of 1.2 kb and 370 bp sized bands (based on where the sgRNA was designed) (Fig. 1b). The Tox3-RNP complex was subsequently transformed into protoplasts of *P. nodorum* through PEG-mediated transformation. More than 50 colonies (transformants) were obtained on plates. Six colonies, which were phenotypically identical to wild type, were randomly chosen and screened by sequencing across the targeted region in *Tox3*. Subsequent sequence analysis identified multiple changes (SNPs and indels) across the targeted region in all mutants confirming an editing efficiency of 100% (Fig. 1c). Note that off target effects were not screened for.

**Fig. 2:**
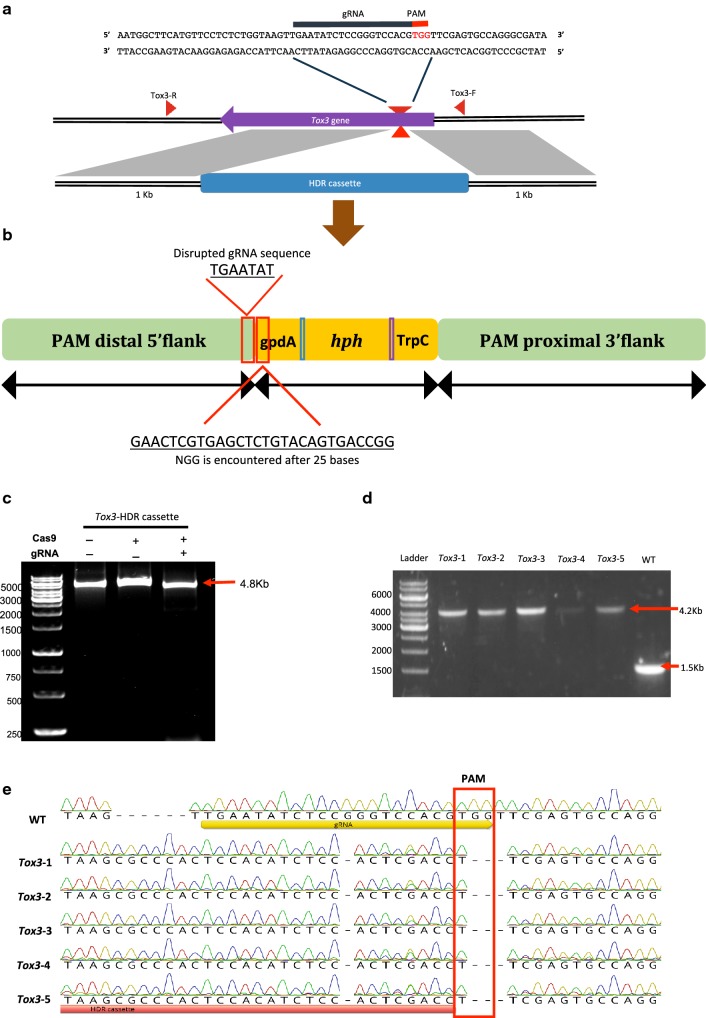
Disruption of *Tox3* gene generated using the *Tox3*-RNP complex and *Tox3*-HDR cassette with 1 kb homologous flanks to *Tox3* gene. **a** A sequence schematic highlighting the sequence of the gRNA and location of the targeted site in the *Tox3* sequence. **b** Design of an HDR cassette with 1 kb homologous flanks on each side. **c** A gel electrophoresis image showing that the *Tox3*-HDR cassette was intact in presence of *Tox3*-RNP complex. **d** PCR screening of 5 selected *Tox3* mutants with 4.2 kb band size (predicting insertion of hygromycin cassette in the target site) and wild type with a band size of 1.5 kb. **e** Multiple sequence alignment of the five mutants screened above demonstrating integration into the expected target cut site

Having proven the efficacy of gene editing using CRISPR/Cas9 in *P. nodorum*, we then explored its utility in facilitating homologous recombination by including an HDR cassette. Although the gene editing described above was very effective, the ability to manipulate genomic DNA through homologous recombination is important for other reverse genetic approaches (e.g. generation of fusion proteins). Current rates for homologous recombination in *P. nodorum* varies from gene to gene, but is reported to be often between 2 and 10% [25]. We were interested to see if the efficiency of recombination with a disruption cassette harbouring homologous flanking DNA could be improved with a CRISPR/Cas9 approach. To test this, a *Tox3*-HDR cassette was constructed consisting of a hygromycin B gene flanked by 1 kb of homologous DNA on either side of the targeted cleavage site in *Tox3* (Fig. 2a). An in vitro cleavage assay of the *Tox3*-HDR cassette demonstrated that it was not susceptible to the RNP complex and therefore suitable for transformation (Fig. 2c). Co-transformation of the *Tox3*-RNP complex with the *Tox3*-HDR cassette yielded forty-four hygromycin-resistant mutants. Initial screening by colony PCR revealed that 70% of the 44 transformants harboured the HDR cassette at the targeted location in Tox3. Genomic DNA was subsequently extracted from five putative positive mutants and re-screened by PCR. Gel electrophoresis revealed an amplification product in the mutants 2.7 kb larger than in the wild type confirming that the HDR cassette was inserted into *Tox3* for 70% of the mutants (Fig. 2d). Successful integration of the HDR cassette into the targeted cut site in *Tox3* was confirmed through sequence analysis of five randomly chosen mutants in comparison to the wild type (Fig. 2e). This result confirms the effective establishment of CRISPR/Cas9-mediated homologous recombination in *P. nodorum*.

**Fig. 3:**
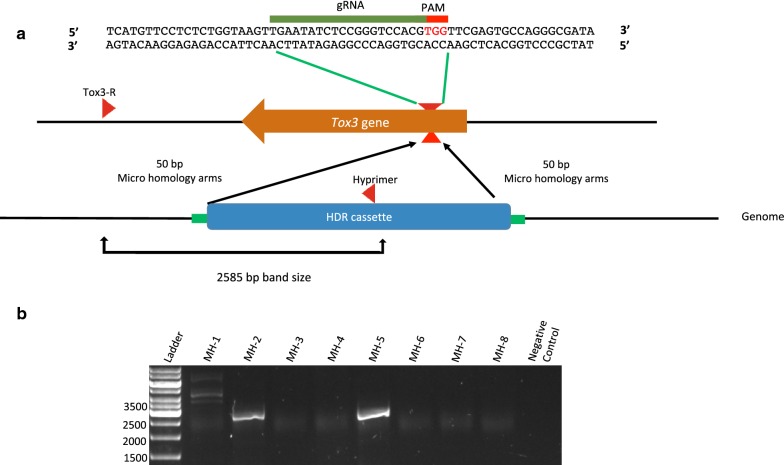
**a** A schematic diagram of *Tox3*-HDR cassette with 50 bp microhomology arms targeting *Tox3* gene. **b** PCR amplification of eight mutant colonies where the mutant (with hygromycin insertion) predicted band size is 2585 bp. The screening primers were designed such that a product will only amplify in the event of homologous recombination (i.e. no product will amplify in the wild-type). MH represents microhomology mutant

Given the success of the Cas9-RNP approach in *P. nodorum* using a HDR cassette with 1 kb homologous arms, we reduced the length of homologous flanking DNA to 50 bp (Fig. 3a). These micro-homology flanks are convenient for construct development as they can be incorporated into primers used to amplify the selectable marker. Using this approach, 50 bp tails homologous to the targeted cleavage site were incorporated into primers used to amplify the hygromycin selectable marker. The resulting HDR cassette (*Tox3*^MH^-HDR) was subsequently co-transformed with the *Tox3*-RNP complex into *P. nodorum* protoplasts. A total of eight colonies growing on the hygromycin selection were screened by PCR of which two were positive for hygromycin in the targeted *Tox3* site (Fig. 3b). Consequently, despite the lower homologous recombination efficiency, the microhomology arms is an efficient approach for marker-based genome editing with the CRISPR/Cas9 system in *P. nodorum*.

## Conclusions

These data demonstrate that CRISPR/Cas9 gene editing is a potent tool for reverse genetic approaches in *P. nodorum*. The efficacy of gene editing in the absence of an HDR cassette provides a powerful option for generating marker-free disruption mutants in *P. nodorum*. Using this approach, there is no need to generate complex gene knockout constructs or rely upon non-homologous end joining pathway mutants [8]. Rather, only a couple of days are required to synthesize and confirm the sequence of the sgRNA before forming the RNP complex with Cas9 prior to transformation. The efficacy in using the HDR cassette, including with use of micro-homology flanks, to facilitate homologous recombination also provides an attractive option when aiming to insert homologous or heterologous DNA at high efficiency and resolution into the *P. nodorum* genome.

## Methods

### Strains used and growth conditions

*Escherichia coli* strain DH5alpha was used for maintenance of plasmids and was routinely grown in LB media (yeast extract, 10 g/L; tryptone, 16 g/L; NaCl, 5 g/L and 20 g/L agar) at 37 °C. *P. nodorum* strain SN15 was grown on V8-PDA (V8 juice, 150 mL/L; PDB, 10 g/L; CaCO_3_ 3 g/L; agar, 15 g/L and pH adjusted to 6) at 22 °C with 12 h of light per day as previously described [26].

### Guide RNA design and cloning

The gene of interest was screened for target sites using NGG as the protospacer adjacent motif (PAM) using Geneious software version 9.1.8. Potential sgRNAs were scored for on-target activity [27] (Additional file 1: Table S1). The best scoring sgRNA sequence was manually checked using BLAST analysis on the reference genome to avoid any potential off-target activity. sgRNA sequences were ordered as oligonucleotides (forward and reverse) with the overhangs having *Bsa*I restriction sites to enable cloning into the DR274 plasmid (Addgene plasmid #42250) [28]. These oligonucleotides were annealed as previously described (https://www.addgene.org/crispr/zhang/). Briefly, 1 μL (100 μM) of each oligonucleotide was combined with 1 μL 10× T4 DNA ligase buffer (New England Biolabs), 6.5 μL ddH_2_O and 0.5 μL T4 Polynucleotide Kinase (New England Biolabs). This reaction was incubated at 37 °C for 30 min prior to incubation at 95 °C for 5 min and then ramping down to 25 °C at 5 °C per min. The annealed oligonucleotides were cloned into *Bsa*I-digested DR274 backbone vector following a slight modification from https://www.addgene.org/crispr/zhang/. Briefly, the PCR duplex was diluted 1:10 instead of 1:200 and incubated with the digested plasmid at room temperature for 30 min. The ligated mixture (2 μL) was transformed into *E. coli* and sgRNA insertion and sequence was confirmed by sequencing of the plasmid with M13F and M13R primers followed by plasmid extraction through Zyppy^Tm^ Plasmid Miniprep Kit (Zymo Research).

### In vitro transcription of sgRNA

The plasmid containing sgRNA was linearized with the *Hind*III restriction enzyme and gel purified. 1 μg of the linearized plasmid was then used as a template for in vitro transcription of the sgRNA using the HiScribe™ T7 High Yield RNA Synthesis Kit (New England Biolabs). The reaction was incubated at 37 °C for 18 h and synthesized gRNA (sgRNA) was purified using Agencourt RNAClean XP cleanup beads (Beckman Coulter). The concentration of in vitro synthesized gRNA was assayed using the Qubit fluorometric quantitation system (Thermo Fisher Scientific) and stored at − 20 °C.

### Purification of the Cas9 protein

The Cas9 protein was purified as previously described [29]. In brief, the pMJ915 plasmid (Addgene plasmid #69090) [30]) which encodes a 6xHistidine (His)—Maltose binding protein (MBP)—Tobacco etch virus cleavage site—Cas9 fusion protein was transformed into Rosetta2 DE3 cells (Merck). Cas9 protein was expressed using the auto-induction method [31]. Protein purification involved Ni-affinity purification, removal of the His-MBP fusion using TEV protease, followed by ion-exchange chromatography and size-exclusion chromatography. Purified Cas9 was concentrated to 10 mg/mL in 20 mM HEPES–KOH pH 7.5, 150 mM KCl, 5% glycerol and 1 mM dithiothreitol, snap frozen in liquid nitrogen and stored at − 80 °C.

### Construction of homology directed repair (HDR) cassettes

The *gpdA* promoter and hygromycin-B resistance gene were amplified from plasmid pAN7-1 using NewPgpdA-F and TtrpC-R [32]. Homologous flanks included in the HDR cassettes were amplified from DNA flanking the sgRNA site in the targeted gene (*Tox3*) (primers are listed in Additional file 2: Table S2). The 5^′^ flank, upstream of the PAM sequence, only included seven bases of sgRNA rather than twenty bases and the HDR cassette encountered an NGG sequence 20–25 bp away from the targeted cut sites avoiding the generation of a potential PAM site in the cassette (Fig. 2b). The HDR cassettes comprising the homologous flanks and selectable markers were then assembled in yeast (*Saccharomyces cerevisiae*) as previously reported [33]. The yeast transformation was conducted using the Frozen-EZ Yeast Transformation II Kit^Tm^ (ZymoResearch) as per manufacturer’s instructions. Yeast colonies harbouring the transformed plasmid were confirmed on selective drop out media (minus uracil). Yeast plasmid extraction was performed using the Zymoprep™ II mini prep protocol (ZymoResearch). Plasmids were then transformed into *E. coli* and subsequently screened by colony PCR. Takara ExTaq was used for all PCR reactions in this study.

### In vitro cleavage assay

The in vitro cleavage assay was modified from a previously described protocol [29]. Briefly, 1 μL of Cas9 protein (1 μg) was mixed with 1.5 μL of sgRNA (1.5 μg) and incubated at room temperature for 10–15 min to form the RNP assembly. After this, 16 μL of 1× cleavage buffer (10 mM MgCl_2_, 0.1 mM EDTA, 20 mM HEPES pH 7.5, 150 mM KCl, 0.5 mM DTT (added fresh while using the buffer) was added to the RNP assembly followed by the addition of 2 μL of PCR product of the targeted gene (100–120 ng). The mixture was centrifuged briefly and incubated immediately at 37 °C for 90 min. If potential non-specific cleavage of an HDR cassette was also being assayed, 100–120 ng of the cassette was also added to the reaction mixture. After the incubation, 3 μL of 0.25 M EDTA and 4 μL of agarose gel electrophoresis loading dye was added to the tube, mixed well and incubated for 10 min at 60 °C. The reaction mixture was then centrifuged at 14,000*g* for 5 min and the supernatant was analysed by gel electrophoresis to visualize the cleaved products.

### Fungal protoplast transformation

*Parastagonospora nodorum* protoplast preparation and transformation was undertaken as previously described [34].

Cas9 (6 μg) and sgRNA (1.5 μg) were assembled at room temperature for 10–15 min to form the ribonuclear protein (RNP) complex [29]. When undertaking the homology directed repair approach, 3–4 μg of PCR amplified HDR cassette was included with RNP complex and transformed into protoplasts.

### Fungal DNA extraction

*Parastagonspora nodorum* mycelia were harvested from agar plates after 12–14 days growth and freeze-dried overnight. A tungsten carbide bead was added and the dried fungal material was ground to a fine powder using a TissueLyserIT (Qiagen) operating for 2 cycles at 50 Hz for 2 min. 300 μL of TE buffer (10 mM Tris, 1 mM EDTA pH8) with 1% SDS was added to the ground spores/mycelia and incubated for 10 min at 50 °C shaking at 350 rpm. After incubation, 300 μL of 2.8 M potassium acetate was added and mixed prior to centrifugation at 14,000*g* for 10 min. The supernatant was transferred to a fresh tube pre-filled with 500 μL of isopropanol, mixed by inversion and then centrifuged at 14,000*g* for 10 min. The resulting DNA pellet was rinsed with 250 μL of 70% ethanol before being briefly air-dried and resuspended in 30 μL of sterile water. The extracted DNA was further diluted 1:100 in water for PCR screening.

## Supplementary information

**Additional file 1.** Table of gRNA sequences.

**Additional file 2.** Table of primer sequences used in this study.

## Data Availability

No substantive datasets were generated during the course of this study and all data is resented within.
