# Supplementary material for: Assessing the efficacy of CRISPR/Cas9 genome editing in the wheat pathogen Parastagonspora nodorum
Source: Fungal Biol Biotechnol. 2020 Mar 31;7:4. doi: 10.1186/s40694-020-00094-0 (PMC7110818; doi:10.1186/s40694-020-00094-0)
Supplement: Supplementary file 1 — Additional file 1. Table of gRNA sequences. [file 40694_2020_94_MOESM1_ESM.docx]

**Table S1**

| **gRNA as Oligonucleotides** | **Sequences** | **Score** |
| --- | --- | --- |
| Tox3-0.831-1 | *TAGG*TGAATATCTCCGGGTCCACG | 0.831 |
| Tox3-0.831-2 | *AAAC*CGTGGACCCGGAGATATTCA |  |

Note: Italicized sequences are overhangs to *Bsa*I restriction site
