# Supplementary material for: Assessing the efficacy of CRISPR/Cas9 genome editing in the wheat pathogen Parastagonspora nodorum
Source: Fungal Biol Biotechnol. 2020 Mar 31;7:4. doi: 10.1186/s40694-020-00094-0 (PMC7110818; doi:10.1186/s40694-020-00094-0)
Supplement: Supplementary file 2 — Additional file 2. Table of primer sequences used in this study. [file 40694_2020_94_MOESM2_ESM.docx]

**Table S2**

| **No.** | **Primers** | **Sequences** |
| --- | --- | --- |
| 1 | TOX3-0.831-1 | *TAGG*TGAATATCTCCGGGTCCACG |
| 2 | TOX3-0.831-2 | *AAAC*CGTGGACCCGGAGATATTCA |
| 3 | TOX3-F | ATTGCAGTTACTGCGCTGTTGCT |
| 4 | TOX3-R | AGCGCAAATCTAAATGCACGCTT |
| 5 | S-TOX3-5'flank-F | CTAAGTAAAACATACTACTT |
| 6 | S-TOX3-3'flank-R | TACATCTGAGTAACACGCT |
| 7 | NewPgpdA-F | GAACTCGTGAGCTCTGTACAG |
| 8 | TtrpC-R | GGTCGAGTGGAGATGTGGAGT |
| 9 | S-TOX3-5'flank-F-PacI-overhang | *GAAACAGCTATGACATGATTACGAATTCTTAAT*CTAAGTAAAACATACTACTT |
| 10 | S-TOX3-5'flank-R-PgpdA overhang | *AGTCACCGGTCACTGTACAGAGCTCACGAGTTC*ATATTCAACTTACCAGAGA |
| 11 | S-TOX3-3'flank-F-trpc overhang | *GTGTAAGCGCCCACTCCACATCTCCACTCGACC*TTCGAGTGCCAGGGCGATA |
| 12 | S-TOX3-3'flank-R-PmeI overhang | *CCGCCAATATATCCTGTCAAACACTGATAGTTT*TACATCTGAGTAACACGCT |
| 13 | 5'-Microhomology-Hyg | *TGGAGAACCATTGAAAATGGCTTCATGTTCCTCTCTGGTAAGTTGAATAT*GAACTCGTGAGCTCTGTACAG |
| 14 | 3'-Microhomology-Hyg | *TCCTCCTTGTTGTCCAAGCAGCTGCGACAGCTATCGCCCTGGCACTCGAA*GGTCGAGTGGAGATGTGGAGT |
| 15 | Hyprimer | TCTCGATGAGCTGATGCTT |

**Note**: Italicized sequences are overhangs designed with primers to be cloned in yeast.

Primer pair Tox3-F and Tox3-R were used to amplify Tox3 gene and to screen mutants.

S-TOX3-5’flank-F and S-TOX3-3’flank-R were used to PCR amplify the HDR cassette along with homologous flanks.

5’flank of HDR cassette was amplified using primer pair S-TOX3-5'flank-F-PacI-overhang and S-TOX3-5'flank-R-PgpdA overhang and 3’flank of HDR cassette was amplified using S-TOX3-3'flank-F-trpc overhang and S-TOX3-3'flank-R-PmeI overhang.

Micro homology arms were incorporated using primer pair 5'-Microhomology-Hyg and 3'-Microhomology-Hyg.

NewPgpdA-F and TtrpC-R primers were used for PCR amplification of HDR cassette including hygromycin, gpdA promoter and trpC terminator.

Tox3-R and Hyprimer were used to screen micro homology mutants.
